# Comparative assessment of fermentative capacity of different xylose-consuming yeasts

**DOI:** 10.1186/s12934-017-0766-x

**Published:** 2017-09-13

**Authors:** Henrique César Teixeira Veras, Nádia Skorupa Parachin, João Ricardo Moreira Almeida

**Affiliations:** 1 Brazilian Agricultural Research Corporation, EMBRAPA Agroenergia, Parque Estação Biológica, PqEB-W3 Norte Final-s/nº, Brasília, DF CEP 70.770-901 Brazil; 2 Graduate Program on Molecular Biology, Department of Cellular Biology, University of Brasília, Campus Darcy Ribeiro, Brasília, DF Brazil; 3 Graduate Program on Chemical and Biological Technologies, Institute of Chemistry, University of Brasília, Campus Darcy Ribeiro, Brasília, DF Brazil

**Keywords:** Xylose fermentation, Xylose reductase, Bioethanol, Yeast fermentation, Oxygen availability

## Abstract

**Background:**

Understanding the effects of oxygen levels on yeast xylose metabolism would benefit ethanol production. In this work, xylose fermentative capacity of *Scheffersomyces stipitis*, *Spathaspora passalidarum*, *Spathaspora arborariae* and *Candida tenuis* was systematically compared under aerobic, oxygen-limited and anaerobic conditions.

**Results:**

Fermentative performances of the four yeasts were greatly influenced by oxygen availability. *S. stipitis* and *S. passalidarum* showed the highest ethanol yields (above 0.44 g g^−1^) under oxygen limitation. However, *S. passalidarum* produced 1.5 times more ethanol than *S. stipitis* under anaerobiosis. While *C. tenuis* showed the lowest xylose consumption rate and incapacity to produce ethanol, *S. arborariae* showed an intermediate fermentative performance among the yeasts. NAD(P)H xylose reductase (XR) activity in crude cell extracts correlated with xylose consumption rates and ethanol production.

**Conclusions:**

Overall, the present work demonstrates that the availability of oxygen influences the production of ethanol by yeasts and indicates that the NADH-dependent XR activity is a limiting step on the xylose metabolism. *S. stipitis* and *S. passalidarum* have the greatest potential for ethanol production from xylose. Both yeasts showed similar ethanol yields near theoretical under oxygen-limited condition. Besides that, *S. passalidarum* showed the best xylose consumption and ethanol production under anaerobiosis.

## Background

Conversion of all sugars present at cellulose and hemicellulose fractions of biomass would increase production and reduce cost of second-generation ethanol [1, 2]. *Saccharomyces cerevisiae* is the main yeast used for alcohol production worldwide, but it cannot produce ethanol from xylose, the second most abundant sugar in nature, unless when genetically engineered [3, 4]. Despite the relative success of engineered strains, recombinant *S. cerevisiae* strains show lower fermentation rates and less tolerance to fermentation inhibitors when fermenting xylose instead of glucose [5, 6]. Thus, the isolation, identification and characterization of native xylose-fermenting yeasts have received great attention in the past years [7–12].

Among the few naturally xylose-fermenting yeasts species, *Scheffersomyces (Pichia) stipitis* is one of the most studied [8, 12, 13]. It has been isolated from the gut of insects and its fermentation capability evaluated in different lignocellulosic hydrolysates [14]. More recently, yeasts from *Spathaspora* and *Candida* genera, as *Spathaspora passalidarum*, *Spathaspora arborariae* and *Candida tenuis*, have been isolated from rotting-wood samples or wood-boring insects and characterized as xylose fermenting yeasts [7, 9, 10]. Like *S. stipitis*, *S. passalidarum* showed xylose fermentation yields above 0.40 g ethanol g^−1^ sugar in both defined and lignocellulosic hydrolyzed medium Slininger [14, 15]. In general, naturally xylose-fermenting yeasts are able to ferment xylose only when the oxygen flow is tightly regulated. High oxygenation level leads to aerobic growth and low ethanol yield, whereas limited dissolved oxygen slows the fermentation rate, increases xylitol accumulation and causes poor ethanol productivity [1, 8, 15–17].

In yeasts, xylose is first reduced to xylitol, a reaction catalyzed by a NAD(P)H-dependent xylose reductase (XR). Then, a NAD^+^-dependent xylitol dehydrogenase (XDH) oxidises xylitol to xylulose [18–20]. Subsequently, xylulose enters into the pentose phosphate and glycolysis pathways, finally being converted to ethanol. Recently, it was shown that *S. passalidarum* exceptionally harbor two XRs, and one of them, preferentially uses NADH as cofactor [20].

As fermentative conditions like media composition, cell density and oxygen availability are usually different [1, 10, 20] a comparative assessment among xylose-consuming yeasts based on literature data becomes difficult. In addition, few studies on physiology of *C. tenuis* and *S. arborariae* are available [10, 20, 21]. Thus, a systematic comparison of fermentative physiology of *S. stipitis*, *S. passalidarum*, *S. arborariae* and *C. tenuis* is still missing and it might help elucidate important steps on xylose metabolism.

The aim of this study was to compare the alcoholic fermentative capacity of four native xylose-consuming yeasts under different oxygenation conditions. The physiology of *S. stipitis*, *S. passalidarum*, *S. arborariae* and *C. tenuis* in defined mineral medium containing xylose as sole carbon source was assessed under aerobic, oxygen-limited and anaerobic conditions. The results presented clearly distinguished the best performing yeast for each condition and highlights the importance of cofactor usage on ethanol production from xylose.

## Methods

### Strains

The yeasts employed in this study were *Scheffersomyces (Pichia) stipitis* NRRL Y-7124, *S. passalidarum* NRRL Y-27907, *S. arborariae* NRRL Y-48658 and *C. tenuis* NRRL Y-1498. All yeasts were preserved in 30% glycerol at −80 °C.

### Xylose fermentations under different oxygen conditions

The xylose fermentation experiments were carried out in bioreactors (Multifors 2, Infors HT) with 500 mL working volume. Cells from −80 °C stock were initially grown in solid YPD medium (10 g L^−1^ yeast extract, 20 g L^−1^ peptone, 20 g L^−1^ glucose), overnight at 28 °C. One single colony was used to inoculate 50 mL of defined mineral medium [22] containing per litre: (NH_4_)_2_SO_4_, 12.5 g; KH_2_PO_4_, 7.5 g; MgSO_4_·7H_2_O, 1.25 g; EDTA, 37.5 mg; ZnSO_4_·7H_2_O, 11.25 mg; MnCl_2_·2H_2_O, 2.5 mg; CoCl_2_·6H_2_O, 0.75 mg; CuSO_4_·5H_2_O, 0.75 mg; Na_2_MoO_4_·H_2_O, 1.0 mg; CaCl_2_·2H_2_O, 11.25 mg; FeSO_4_·7H_2_O, 7.5 mg; H_3_BO_3_, 2.5 mg; KI, 0.25 mg. Filter-sterilized vitamins were added after heat sterilization of this medium. Final vitamin concentrations per litre were: biotin, 0.125 mg; Ca-pantothenate 2.5 mg; nicotinic acid 2.5 mg; inositol 62.5 mg; thiamin-HCl 2.5 mg; pyridoxine–HCl 2.5 mg; *P*-aminobenzoic acid 0.5 mg; riboflavin 0.5 g and; folic acid 0.005 g. The carbon source consisted of 40 g L^−1^ xylose.

The start culture at the bioreactor was OD_600 nm_ of 0.5. Cultures were maintained with pH 5.5, by addition of KOH 3 M, under agitation—stirrer at 400 rpm, and temperature of 28 °C. First, yeast performance was evaluated under aerobic and oxygen-limited conditions. For aerobic experiments, synthetic air (20% pure oxygen and 80% pure nitrogen) was injected in the reactor at 0.5 L min^−1^. The dissolved oxygen measured in the reactor (Sensors METTLER TOLEDO) was above 60% during the entire fermentation period. For oxygen-limited experiments, the airflow of synthetic air was adjusted for 0.05 L min^−1^, which resulted in dissolved oxygen below 10% in the first 10 h of fermentation and zero afterwards. But the airflow was kept constant in a minimal rate, indicating that the entire oxygen that was entering the bioreactor was promptly consumed. All fermentations were carried out in biological triplicates.

Anaerobic fermentations with the four yeasts were performed in small cap vials sealed with a rubber stopper, equipped with a needle for carbon dioxide removal. Cells from −80 °C stock were initially grown in solid YPD medium, overnight at 28 °C. One single colony was used to inoculate 50 mL of defined mineral medium as described above. The culture started with a high cell density equal to OD_600 nm_ of 2.0. The pH was adjusted for 5.5 and, the flasks incubated under agitation—stirrer at 400 rpm and temperature of 28 °C. All experiments were carried out in biological triplicates.

### Analytical methods

To monitor yeast growth, samples were withdrawn regularly during fermentations and biomass was measured by optical density at 600 nm using a spectrophotometer (SpectraMax^®^ M3, Molecular Devices). For cell dry weight (CDW) measurement, 5 mL of pre-inoculum culture and of the stationary phase of the growth during all fermentations were withdrawn and centrifuged (12,000×*g*, 5 min). Before weighing, the pellet was incubated in glass tube for at least 48 h at 60 °C. The cell dry weight was correlated with OD_600 nm_ measured in the same time intervals. Each measurement was performed in duplicate.

Sugar consumption and products formed during fermentation experiments were measured using a high-pressure liquid chromatograph (HPLC) system. Initially, samples withdrawn regularly during fermentations were centrifuged (12,000×*g*, 5 min) and the supernatant was transferred to a new tube. Concentrations of xylose, xylitol, glycerol, acetate and ethanol in supernatants were measured using HPLC system (Acquity UPLC H Class, Waters) equipped with a refractive index detector and an Aminex^®^ HPX-87H column (Bio-Rad) at 45 °C. The mobile phase was 5 mM sulfuric acid at a flow rate of 0.6 mL min^−1^. Results are shown as average ± standard deviations.

### Enzymatic assays for XR and XDH

The enzymatic activity of xylose reductase (XR) and xylitol dehydrogenase (XDH) in crude-cell extracts was measured according to [23]. For this, 5 mL of cell suspension of *S. stipitis*, *S. passalidarum*, *S. arborariae* and *C. tenuis* were collected in the middle of the exponential growth phase during aerobic and oxygen-limited fermentations. Cells were pelleted by centrifugation, washed with sterile water, and lysed with Y-PER^®^—Yeast Protein Extraction Reagent (Pierce, Rockford, USA) to obtain cell-crude extracts. Protein concentrations in cell-free extracts were determined using Quick StartTM Bradford Protein Assay Kit (Bio-Rad Laboratories Ltda., USA), following the manufacture’s instruction.

XR reaction mixture contained 100 mM triethanolamine buffer (pH 7.0), 0.2 mM NADH or NADPH, 350 mM xylose. XDH reaction contained 100 mM triethanolamine buffer (pH 7.0), 0.3 mM NAD^+^, 300 mM xylitol. All reactions were started with addition of limiting substrates. The assays were performed at 30 °C and the oxidation of NADH/NADPH and reduction of NAD^+^ were followed as the change in absorbance at 340 nm. The value of 6.22 mL (μmol cm)^−1^ was used as the molar absorption coefficient of coenzymes per minute. The specific activities of XR and XDH were given in units per mg protein (U mg^−1^). Enzyme unit is defined as 1 μmol of cofactor reduced or oxidized per minute. All assays were performed in triplicate and the results are shown as means ± standard deviations.

## Results and discussion

### Xylose fermentation in defined mineral medium

**Fig. 1:**
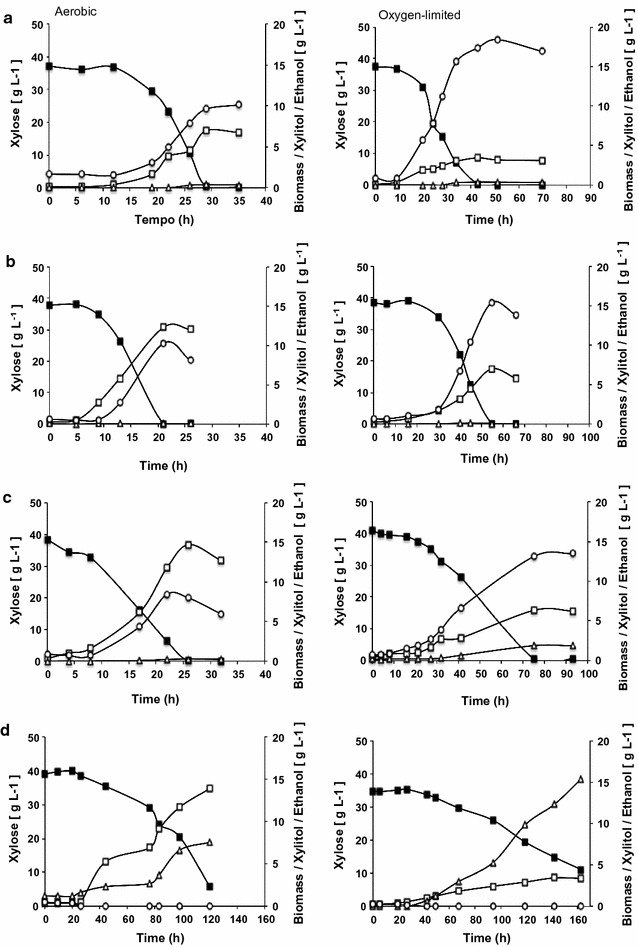
Xylose fermentation under different oxygen level conditions. Left: aerobic and right: oxygen-limited. *S. stipitis* (**a**); *S. passalidarum* (**b**); *S. arborariae* (**c**) and; *C. tenuis* (**d**). Xylose (closed square), biomass (open square), xylitol (open triangle) and ethanol (open circle). The different scales on x-axis highlight different fermentation rates

The fermentative capacity of the four naturally xylose-consuming yeasts, *S. stipitis*, *S. passalidarum*, *S. arborariae* and *C. tenuis* were evaluated under aerobic, oxygen-limited and anaerobic conditions. Xylose consumption varied considerably among the four yeasts both under aerobic and oxygen-limited conditions (Fig. 1). *Scheffersomyces stipitis*, *S. passalidarum* and *S. arborariae* were able to consume xylose completely and showed at least 6 times higher specific xylose consumption rate than *C. tenuis* under aerobic condition (Fig. 1; Table 1). Even after more than 120 h of fermentation, *C. tenuis* consumed about 34 and 28 g L^−1^ of xylose under aerobic and oxygen-limited conditions, respectively (Fig. 1d). When cultivated under oxygen limitation, *S. stipitis* and *S. passalidarum* showed similar xylose consumption rates, however the rate was 2 times lower for *S. arborariae* (Table 1). As expected, biomass yields for all yeasts were 2 times higher under aerobic than oxygen-limited condition. However, biomass yield was twofolds higher for *S. passalidarum* than *S. stipitis*. This may explain the lower ethanol productivity rate for this yeast compared to *S. stipitis* under aerobic condition (Table 1).

**Table 1:** Parameters calculated for xylose fermentation

Yeasts species	Oxygen condition	Xylitol (g L^−1^)	Ethanol (g L^−1^)	Xylitol yield [Y_x/s_ (g g^−1^)]	Ethanol yield [Y_e/s_ (g g^−1^)]	Biomass yield [Y_b/s_ (g g^−1^)]	Specific xylose consumption [(g g_cdw_^−1^ h^−1^)]	Specific ethanol productivity [(g g_cdw_^−1^ h^−1^)]
*S. stipitis* *S. passalidarum* *S. arborariae* *C. tenuis*	Aerobic	0.41 ± 0.06	8.05 ± 0.91	0.01 ± 0.00	0.24 ± 0.02	0.16 ± 0.04	0.30 ± 0.09	0.08 ± 0.03
		0.04 ± 0.00	10.06 ± 0.48	0.00 ± 0.00	0.28 ± 0.02	0.33 ± 0.02	0.13 ± 0.02	0.04 ± 0.01
		0.27 ± 0.08	8.65 ± 1.16	0.01 ± 0.00	0.25 ± 0.02	0.31 ± 0.05	0.13 ± 0.02	0.03 ± 0.01
		8.03 ± 1.48	0.00 ± 0.00	0.30 ± 0.06	0.00 ± 0.00	0.43 ± 0.06	0.02 ± 0.00	0.00 ± 0.00
*S. stipitis* *S. passalidarum* *S. arborariae* *C. tenuis*	Oxygen- limited	0.37 ± 0.01	16.48 ± 0.83	0.01 ± 0.00	0.45 ± 0.04	0.09 ± 0.02	0.29 ± 0.09	0.10 ± 0.02
		0.05 ± 0.02	16.36 ± 1.40	0.00 ± 0.01	0.44 ± 0.04	0.13 ± 0.04	0.22 ± 0.10	0.10 ± 0.05
		1.82 ± 0.66	11.47 ± 2.37	0.04 ± 0.02	0.31 ± 0.02	0.15 ± 0.01	0.09 ± 0.01	0.03 ± 0.01
		15.43 ± 1.90	0.00 ± 0.00	0.62 ± 0.04	0.00 ± 0.00	0.14 ± 0.01	0.04 ± 0.01	0.00 ± 0.00

The fermentative capacities were measurement under aerobic and oxygen-limited conditions. The values are calculated considering the exponential growth phase

**Fig. 2:**
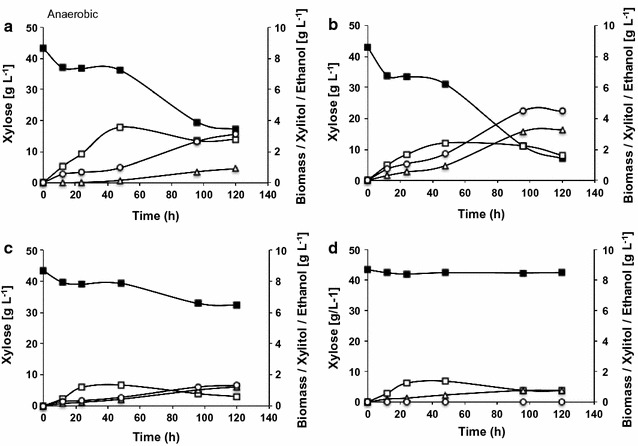
Xylose fermentation under anaerobic condition. *S. stipitis* (**a**); *S. passalidarum* (**b**); *S. arborariae* (**c**) and; *C. tenuis* (**d**). Xylose (closed square), biomass (open square), xylitol (open triangle) and ethanol (open circle)

Growth and ethanol production by these yeasts were strongly influenced by the oxygen availability. Biomass formation was favored in presence of oxygen whereas ethanol production was favored under more strictly oxygen availability. Produced biomass varied from a minimal of 6.7 g L^−1^ for *S. stipitis* to a maximum of 13.9 g L^−1^ to *C. tenuis* under aerobic condition (Fig. 1). Despite the variation in the total biomass, the specific growth rate of *S. stipitis*, *S. passalidarum* and *S. arborariae* ranged from 0.15 to 0.18 h^−1^. This is about 2 times higher than *C. tenuis* under aerobic conditions, which reached specific growth rate 0.08 h^−1^. The biomass formation was about 2 times lower under oxygen-limited condition than in aerobic one, varying from 3.0 to 7.0 g L^−1^ (Fig. 1). In anaerobic condition the maximum biomass formation reached was 3.5 g L^−1^ for *S. stipitis*, 2.4 g L^−1^ for *S. passalidarum*, 1.3 g L^−1^ for *S. arborariae* and 1.2 g L^−1^ for *C. tenuis* (Fig. 2).

*Scheffersomyces stipitis*, *S. passalidarum* and *S. arborariae* produced predominantly ethanol from xylose (Fig. 1a–c). Oxygen limitation increased ethanol production and yields by *S. stipitis*, *S. passalidarum* and *S. arborariae*. Indeed, the concentrations increased approximately twofold when compared to aerobic condition (Table 1). *Scheffersomyces stipitis* and *S. passalidarum* reached the highest ethanol yields (0.45 and 0.44 g g^−1^) among the four xylose-consuming yeasts employed in this study (Table 1). These values are in good agreement with previous studies, which showed ethanol yields varying from 0.40 to 0.48 g g^−1^ to *S. stipitis* [16, 24] and from 0.43 to 0.48 g g^−1^ to *S. passalidarum* [17, 20] under limited oxygenation levels. In turn, ethanol yield for *S. arborariae* was only of 0.31 g g^−1^ (Table 1), which was also observed previously in an independent study [20]. Xylitol, glycerol and acetate formation by *S. stipitis*, *S. passalidarum* and *S. arborariae* was minimal and did not show significant differences among them (Fig. 1; Table 1). On the other hand, *C. tenuis* did not produce ethanol under any condition evaluated. Indeed, it produced mainly xylitol during fermentation under oxygen-limited condition (0.62 g g^−1^) (Table 1).

The xylose consumption rate was lower under anaerobic condition in all evaluated yeasts (Fig. 2). Only *S. stipitis* and *S. passalidarum* were able to produce ethanol, and even so, xylitol formation also increased when compared to other aerobic and oxygen-limited conditions (Figs. 1a, b, 2a, b). Insufficient oxygen rate was reported to increase xylitol accumulation and to cause poor ethanol productivity in *S. stipitis* and *S. passalidarum* [17]. Despite the similar fermentative performances of *S. stipitis* and *S. passalidarum* under oxygen-limited condition, *S. passalidarum* consumed more xylose and produced 50% more ethanol than *S. stipitis* in anaerobic condition (Fig. 2a, b). These results are in agreement with those observed in previous work, when *S. passalidarum* showed efficient conversion of xylose into ethanol under anaerobic condition, while the *S. stipitis* almost did not ferment xylose [1]. Another study that assessed the aeration effect on xylose fermentation also showed that *S. passalidarum* (ethanol yield 0.43 g g^−1^) is a better native xylose-fermenting yeast than *S. stipitis* (ethanol yield 0.39 g g^−1^) when a smaller oxygen transfer rate is employed [17].

Although it has been proposed that *C. tenuis* is capable of fermenting xylose [10], it showed the poorest xylose consumption rates among the four yeasts assessed and it was not able to produce ethanol in any condition evaluated in this study (Figs. 1, 2). In the previous work, Wohlbach et al. [10] showed that *C. tenuis* produced approximately 2.0 g L^−1^ ethanol during microaerobic fermentation with 8% xylose and high initial cell density (OD_600 nm_ of 10) in an Erlenmeyer flask. In our study, some change of parameters may have influenced the metabolism of *C. tenuis*, so the xylitol formation by this yeast was significant (up to 15.4 g L^−1^) and ethanol was not detected (Figs. 1, 2; Table 1). The approximately 20 times lower initial cell density (OD_600 nm_ of 0.5, equal to 0.2 g L^−1^), the low flow air rate during oxygen-limited fermentation and the usage of defined mineral medium instead of yeast extract and peptone may have hampered ethanol detection in this work.

### Xylose reductase (XR) and xylitol dehydrogenase (XDH) activities

Xylose reductase (XR) and xylitol dehydrogenase (XDH) activities were measured in crude-cell extracts of *S. stipitis*, *S. passalidarum*, *S. arborariae and C. tenuis* from cells growing under aerobic and oxygen-limited conditions. *S. stipitis*, *S. passalidarum* and *S. arborariae* presented NADH and NADPH-dependent XR activity, whereas *C. tenuis* XR were strictly NADPH-dependent (Table 2). While *S. stipitis* and *S. arborariae* showed higher NADPH-dependent XR activity, *S. passalidarum* showed approximately 1.5 times higher NADH-dependent XR activity.

**Table 2:** Xylose reductase (XR) and xylitol dehydrogenase (XDH) specific activities in crude-cell extracts of *S. stipitis*, *S. passalidarum*, *S. arborariae* and *C. tenuis*

Yeasts species	Oxygen conditions	XR (U mg^−1^)			XDH (U mg^−1^)
		NADH	NAD(P)H	Ratio_NADH/NAD(P)H_	NAD^+^
*S. stipitis* *S. passalidarum* *S. arborariae* *C. tenuis*	Aerobic	0.17 ± 0.06	0.23 ± 0.05	0.74 ± 0.13	0.23 ± 0.11
		2.96 ± 0.40	2.15 ± 0.12	1.38 ± 0.16	0.30 ± 0.05
		0.88 ± 0.13	3.10 ± 0.29	0.29 ± 0.05	0.65 ± 0.07
		–	0.35 ± 0.07	–	0.25 ± 0.05
*S. stipitis* *S. passalidarum* *S. arborariae* *C. tenuis*	Oxygen-limited	0.26 ± 0.07	0.45 ± 0.11	0.59 ± 0.16	0.19 ± 0.08
		0.60 ± 0.08	0.46 ± 0.04	1.29 ± 0.07	0.21 ± 0.03
		0.77 ± 0.17	1.86 ± 0.25	0.43 ± 0.16	0.12 ± 0.04
		–	0.27 ± 0.05	–	0.28 ± 0.06

Yeasts were grown under aerobic and oxygen-limited conditions and samples were withdrawn in the middle of exponential growth phase

The fermentative performances of yeasts under oxygen-limited condition could be directly correlated with the capability to use NADH on xylose reduction (Tables 1, 2). Indeed, *S. passalidarum* showed the highest ratio of NADH/NADPH XR activity (around 1.30) and the best fermentative performance, i.e. higher xylose consumption rate and higher concentration of ethanol, under anaerobic condition; followed by *S. stipitis* and *S. arborariae* with ratios around 0.6 and 0.4, respectively (Table 2). *Candida tenuis*, which XR prefers 33-fold NADPH over NADH [25] did not produce ethanol at all. Accordingly, it was recently shown that *S. passalidarum* possess two XR (genes XIL1.1 and XIL1.2) and one of them uses preferentially NADH as cofactor [20].

XR NADH-preference was previously correlated with improved ethanol production by engineered *S. cerevisiae* strains [18, 26, 27]. The usage of NADH on xylose reduction is advantageous because the redox balance in the xylose catabolic pathways is optimized, since XDH, the next enzyme in the pathway, is strictly NAD^+^-dependent (Table 2) [18]. The NAD^+^ surplus regenerated during xylose reduction would reduce xylitol formation due to higher xylose consumption rate, which impact positively the ethanol yield and productivity [20, 28]. Indeed, strategies aiming to increase NAD^+^ availability during fermentation increases xylose consumption rate and ethanol production. These include mutations to alter cofactor preference of XRs from NADPH to NADH [29], addition of external electron donor [30] or expression of additional reactions that generated increased NAD^+^ availability [31]. No enzymatic activity was performed in the anaerobic condition because the growth was very slow and there was no exponential growth phase. Thus, only fermentative capacity was compared.

## Conclusion

The comparative assessment of the four-native xylose-consuming yeasts showed that the *S. stipitis* and *S. passalidarum* have the greatest potential for ethanol production from xylose. Both yeasts showed similar ethanol yields near theoretical under oxygen-limited condition. Besides that, *S. passalidarum* showed the best xylose consumption and ethanol production under anaerobiosis. The better performing yeasts, i.e. with higher xylose consumption rate and higher concentration of ethanol, during anaerobic xylose showed higher ratio of NADH/NADPH XR activity.
